# The Wisconsin Card Sorting Test: Split-Half Reliability Estimates for a Self-Administered Computerized Variant

**DOI:** 10.3390/brainsci11050529

**Published:** 2021-04-21

**Authors:** Alexander Steinke, Bruno Kopp, Florian Lange

**Affiliations:** 1 Department of Neurology, Hannover Medical School, Carl-Neuberg-Straße 1, 30625 Hannover, Germany; steinke.alexander@mh-hannover.de (A.S.); kopp.bruno@mh-hannover.de (B.K.); 2 Behavioral Engineering Research Group, KU Leuven, Naamsestraat 69, 3000 Leuven, Belgium

**Keywords:** Wisconsin Card Sorting Test, executive functions, neuropsychological assessment, computerized assessment, reliability generalization, split-half reliability

## Abstract

Self-administered computerized assessment has the potential to increase the reach of neuropsychological assessment. The present study reports the first split-half reliability estimates for a self-administered computerized variant of the Wisconsin Card Sorting Test (WCST), which is considered as a gold standard for the neuropsychological assessment of executive functions. We analyzed data from a large sample of young volunteers (*N* = 375). Split-half reliability estimates for perseveration errors, set-loss errors, and inference errors were all above 0.90. Split-half reliability estimates for response time measures on switch and repeat trials exceeded 0.95. Our results indicated sufficient split-half reliability for a self-administered computerized WCST, paving the way for an advanced digital assessment of executive functions. We discuss potential effects of test formats, administration variants, and sample characteristics on split-half reliability.

## 1. Introduction

The reach of neuropsychological assessment is limited by its conventional assessment set-ups that typically require face-to-face expert-administration of paper-and-pencil tools. Self-administered online assessment addresses these efficiency constraints by allowing examinees to complete computerized tools remotely without expert supervision. Self-administered online assessment and related approaches to remote neuropsychological assessment (e.g., teleneuropsychology) have received increasing interest in recent years [1,2,3,4,5,6,7,8]. The COVID-19 pandemic, which poses a potential threat to examinees and examiners in conventional assessment set-ups, is likely to intensify this development [9,10,11].

Progress with regard to the practical implementation of self-administered online assessment depends on the translation of well-established assessment tools into a suitable format [2,5,12]. The present study addresses two translation steps that are crucial for the practical implementation of self-administered online assessment. First, assessment tools need to be converted from traditional paper-and-pencil format into computerized format, enabling examinees to use these tools by means of their own electronic devices. Second, assessment tools must provide sufficient information for successful self-administration. Ideally, these translation steps are completed in a way that retains the psychometric properties of the original neuropsychological tool [6,7,12,13,14]. The present study is concerned with the Wisconsin Card Sorting Test (WCST) [15,16], which is probably the most frequently used neuropsychological tool for the assessment of executive functions [17,18,19]. We examine split-half reliability of speed and accuracy measures of a self-administered computerized WCST variant. We collected data from a sample of young individuals, who were group-wise tested in the lab. Hence, the present study presents a step along the way towards fully remote neuropsychological assessment.

The WCST presents participants with four key cards that depict one red triangle, two green stars, three yellow crosses, and four blue circles. Participants have to sort stimulus cards to key cards according to the color, the shape, or the number of depicted objects. Following any card sort, participants receive positive or negative feedback indicating whether he or she was right or wrong. Successful performance on the WCST requires participants to establish abstract cognitive sets, i.e., to sort stimulus cards by the color, shape, or number category [20]. Participants are supposed to switch the applied cognitive set following negative feedback. In contrast, participants are supposed to maintain the applied cognitive set following positive feedback. 

**Figure 1:**
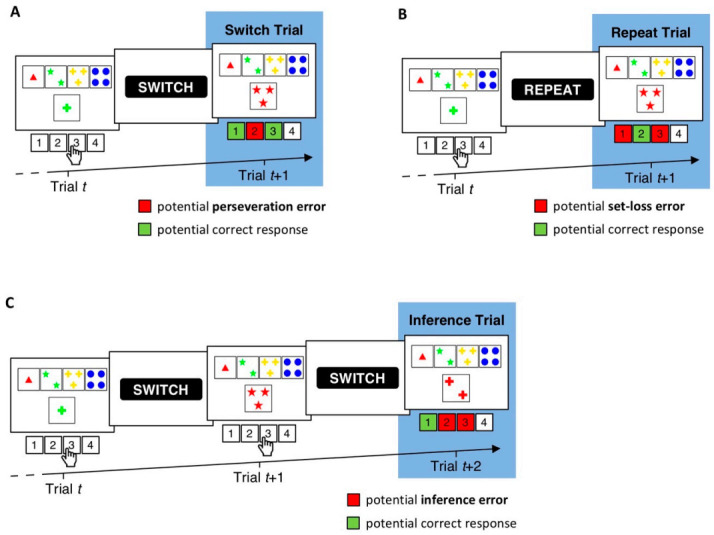
Exemplary trial sequences on a computerized variant of the WCST (i.e., the cWCST) [25]. (**A**). The stimulus card on Trial *t* depicts one green cross. Applicable cognitive sets are the number category (far left key card, response 1), the color category (inner left key card, response 2), and the shape category (inner right key card, response 3). The execution of response 3 indicates the application of the shape category. A succeeding negative feedback cue (i.e., “SWITCH”) indicates that response 3 was incorrect and that the shape category should be switched on the upcoming trial. Trial *t* + 1 therefore constitutes a switch trial. Perseveration errors are erroneous repetitions of the applied cognitive set on switch trials (e.g., the shape category on trial *t* + 1). (**B**). Trial *t* depicts the same stimulus card and response as in A. However, the application of the shape category is now followed by a positive feedback cue (“REPEAT”), indicating that the execution of response 3 was correct and that the shape category should be repeated on the upcoming trial. Set-loss errors are erroneous switches of the applied cognitive set on repeat trials (e.g., the color or number category on Trial *t* + 1). (**C**). On Trial *t*, the execution of response 3 indicates the application of the shape category, which is followed by a negative feedback cue. The execution of response 3 on Trial *t* + 1 indicates a switch to the number category. A subsequently presented negative feedback cue indicates that response 3 was incorrect and that the number category should also be switched. On trial *t* + 2, the participant received all necessary information to infer the prevailing category (i.e., sorting by the shape and the number category were incorrect; thus, the application of the color category must be correct). Hence, trial *t* + 2 constitutes an inference trial [22,23,24]. Failures to infer the prevailing cognitive set (as indicated by the application of any other category than color on trial *t* + 2) are scored as inference errors.

The WCST provides a number of performance indices that were designed to tap different aspects of executive functions [20,21,22]. The number of perseveration errors (PE) refers to the number of failures to shift the applied cognitive set on trials following negative feedback (i.e., switch trials). The number of set-loss errors (SLE) refers to the number of failures to maintain a cognitive set on trials following positive feedback (i.e., repeat trials). Moreover, the number of inference errors (IE) has been introduced as an additional type of error that can provide incremental information about participants’ performance [22,23,24]. The number of IE refers to the number of failures to infer the prevailing category when participants received all necessary information to do so (i.e., on inference trials, which are a sub-type of switch trials). Figure 1 gives illustrative examples of switch, repeat, and inference trials.

There are several well-established manual WCST variants [21,25,26,27,28] that require conventional face-to-face expert-administration. On manual WCST variants, the examiner presents participants with concrete key and stimulus cards that are placed in front of the participant. Participants indicate card sorts by moving stimulus cards next to the intended key card. The examiner provides participants with verbally uttered feedback (i.e., ‘correct’ or ‘incorrect’) following any card sort. Test lengths differ between manual WCST variants, ranging from the completion of 48 trials [26,27] to the completion of 128 trials [21]. Test instructions are verbally provided by the examiner (for an example, see [20]).

There are also a number of computerized WCST variants [29,30,31,32]. The present study is concerned with the cWCST [25]. The cWCST presents participants with target displays on a computer screen. Target displays consist of key cards that appear invariantly above stimulus cards. Participants indicate their card sorts by pressing one of four keys on a conventional computer keyboard that spatially map key card positions. Following any key press, participants receive a visual feedback cue (i.e., “SWITCH” or “REPEAT” [23,25,30,31]). The cWCST requires participants to complete an increased number of trials when compared to common manual WCST variants. The cWCST ends after the completion of 40 switches of the prevailing sorting category (around 160 trials) or the completion of a maximum of 250 trials. In contrast to manual WCST variants, the cWCST provides participants with test instructions on the computer screen. Figure 1 depicts an illustrative example of a trial sequence on the cWCST.

The cWCST can be executed on personal computers. Moreover, the cWCST is designed in a way that should enable participants to successfully self-administer that test. Hence, the cWCST may be suitable for a self-administered online assessment of executive functions. The cWCST also offers a number of additional advantages over manual WCST variants. For example, the cWCST allows to complement the assessment of error scores (e.g., the number of PE, SLE, and IE) along with response time (RT) measures, providing valuable information about participants’ performance [23,33,34,35,36]. The high trial number of the cWCST also renders robust implementation of computational modeling possible [24,37,38,39,40]. Against this background, the cWCST could provide an advanced understanding of executive functions that remains difficult to achieve by manual WCST variants.

However, the digital format of the cWCST, as well as self-administration, may exert effects on its reliability and validity [2,5,6]. The present study addresses the reliability of the cWCST when self-administered. Sufficient reliability is crucial for any clinical test administration because it determines the confidence that one can have in its results at an individual level [20,41,42]. A low reliability renders any interpretation of measures difficult as it remains indistinguishable whether the obtained results are robust or largely arise from measurement error [41,42,43,44].

There are only a few studies that reported reliability estimates for manual WCST measures [45], and there are even less studies that reported reliability estimates for computerized WCST measures [46,47,48,49]. These studies almost entirely used conventional face-to-face expert-administration set-ups. Moreover, these studies mainly reported test–retest reliability estimates for WCST measures. Test–retest reliability estimates are obtained by correlating data from two distinct WCST administrations that were separated by relatively long-lasting time intervals (ranging from less than a day to 30 months [45]). A review of reliability studies of manual WCST variants [45] indicated an across-studies average test–retest reliability estimate of 0.56 for the number PE and 0.16 for failures to maintain set (which roughly correspond to the number of SLE). Test–retest reliability estimates for the number of PE and SLE on computerized WCST variants seem to be comparable in size [46]. These test–retest reliability estimates are far from values which are considered as the desirable minimum (i.e., *r* = 0.90; see page 265 of [44]), putting the conception of WCST measures as reliable indicators of executive functions into doubt [50].

In a recent study [20], we argued that test–retest reliability estimates are not informative with regard to most contexts in which the WCST has been applied. Test–retest reliability estimates refer to the temporal stability of a measure. However, most frequently, the temporal stability of WCST measures is of less importance, rendering estimates of the internal consistency reliability more appropriate and informative. Split-half reliability estimates are common indicators of internal consistency reliability [42,51,52]. Split-half reliability estimation is applicable whenever a measurement consists of repeatedly administered trials (or items [20,53]). The total number of trials of a test is divided into two discretionary construed test halves, such as the first and second test halves or subsets of trials with odd and even trial numbers. Performance indices are calculated for each test half (e.g., by counting the number of PE on the first and second test half). The correlation between performance indices on test halves, when corrected for test length, gives an estimate of a measure’s split-half reliability [42,51,52]. Note that reliability estimates may also be obtained by computing intra-class correlation coefficients. However, this method is of no further interest for the present study.

There are many different options to split a test into halves, and there is an equal number of available split-half reliability estimates for a single measure [20,41,53]. For example, researchers may choose to report split-half reliability estimates obtained from first/second half splits. Alternatively, researchers could also choose to report typically higher estimates obtained from splitting the test by odd/even trial numbers [54,55]. Sampling-based approaches to split-half reliability allow to assess the bias that is associated with such arbitrary test splits [20,41,53,56,57,58,59,60]. Sampling-based split-half reliability estimates are computed by iteratively applying random test splits. For any random test split, a split-half reliability estimate is computed. The resulting frequency distribution of all sampled split-half reliability estimates is informative about potential biases toward higher or lower ends of the range of all achievable split-half reliability estimates. The median of the resulting frequency distribution is furthermore considered as an unbiased estimate of a measure’s split-half reliability [53].

In a recent study [20], we investigated sampling-based split-half reliability estimates for manual WCST measures in a conventional face-to-face expert-administration set-up. Median sampling-based split-half reliability was 0.92 for the number of PE and 0.69 for the number of SLE in a sample of neurological inpatients. These split-half reliability estimates are considerably higher than the test–retest reliability estimates reported by previous studies [45]. Thus, the number of PE (and to a lesser degree the number of SLE) on this manual WCST variant seem to exhibit adequate split-half reliability in samples of neurological inpatients in conventional assessment set-ups.

To our knowledge, there are no investigations of split-half reliability of the cWCST yet. Our present study aimed at closing this gap by reporting the first split-half reliability estimates for the cWCST when self-administered in a laboratory. We report split-half reliability estimates for common error scores (i.e., the number of PE, SLE, and IE) as well as for typical cWCST RT measures (i.e., mean RT on switch, repeat, and inference trials; see Figure 1). We investigated a large sample drawn from the population of young volunteers, which have been frequently studied by computerized WCST variants [23,25,29,31,61,62]. We calculated split-half reliability estimates from systematic test splits (i.e., first/second half split and odd/even split) as well as from iteratively sampled random test splits.

## 2. Materials and Methods

### 2.1. Data Collection

A sample of *N* = 407 participants (155 male, two preferred not to say; *m* = 23.47 years; *SD* = 4.83 years) completed the cWCST while participating in one of the two studies reported by Lange and Dewitte [25]. Participants were recruited from the subject pool of the Faculty of Economics and Business at KU Leuven, Belgium. The majority of participants (90%) were students and indicated to speak Dutch as their first language (66%), but as the subject pool also included many international students, all study materials were administered in English. We excluded 32 participants because of invalid test performance. We considered test performance as invalid when any category was more frequently or less frequently applied than the overall mean of applications of that category plus/minus three standard deviations. The final sample included *N* = 375 participants (144 male, one preferred not to say; *m* = 23.17 years; *SD* = 4.37 years). For details of data collection, see [25].

### 2.2. cWCST

Participants indicated card sorts by executing one of four responses. Response keys spatially mapped the position of key cards (i.e., one red triangle, two green stars, three yellow crosses, and four blue circles). A positive or negative visual feedback cue (“REPEAT” or “SWITCH”, respectively) followed any response execution. Feedback cues appeared 500 ms after response execution and remained on screen for 300 ms. Previous studies in non-clinical populations (e.g., [23,63]) revealed this presentation to be sufficient for feedback-cue perception. The next stimulus card and key cards appeared 500 ms after feedback cue offset and remained on screen until response detection. The prevailing category switched unpredictably following runs of two or more repetitions of the same sorting category. Participants completed a short practice block prior to the experimental block, which ended after six switches of the prevailing category. The experimental block ended after 40 switches of the prevailing category or the completion of 250 trials (including six practice runs). Participants were presented with test instructions before the first practice run. That is, participants received information about the viable categories and about the fact that the prevailing category periodically switches. Test instructions are available from www.osf.io/3ny95. The cWCST was programmed by means of OpenSesame [64] and it is also available from www.osf.io/3ny95.

We tested participants in groups of up to six people. Upon entering the laboratory, the experimenter instructed each group of participants to leave any potentially distracting materials (e.g., cell phones) in the entrance area of the laboratory before directing each participant to a partially enclosed testing cubicle. In the testing cubicle, the cWCST and all task instructions were administered on a personal computer. We offered participants the possibility to contact the experimenter in case of questions, but only few participants made use of this option. When contacted, the experimenter solely repeated the instructions that had been provided on the computer screen. During the assessment, the experimenter took a seat in the entrance area, about 5–10 m away from the different testing cubicles and out of sight of all participants.

We considered three trial types for reliability analysis: switch trials, repeat trials, and inference trials [23]. Figure 1 gives illustrative examples of these trial types. Switch trials are any trials that follow negative feedback. On switch trials, participants committed a PE by repeating the previously applied category. Repeat trials are any trials that follow positive feedback. On repeat trials, participants committed a SLE by switching the previously applied category. Inference trials are a sub-type of switch trials. Inference trials are any trials that follow a switch trial on which the applied category was switched but again followed by a negative feedback. As, after a switch trial, the correct sorting category does not change before it has been identified, participants have all necessary information to infer the prevailing category on inference trials (see Figure 1 for an example). They have received negative feedback for applying two of the three sorting rules, so they can infer that the third sorting rule must now be correct. Participants committed an IE by applying any other category than the prevailing category on an inference trial. For any participant, we computed the number of committed PE, SLE, and IE.

For RT analysis, we excluded all trials with RT faster than 100 ms or RT slower than an individual RT cut-off. We defined the individual RT cut-off as three individual standard deviations above the mean RT of that participant [23]. We also excluded all trials on which participants committed a PE, an SLE, or an IE. For the remaining trials, we computed individual mean RT on switch, repeat, and inference trials. Trial exclusion criteria for RT analysis were consistent with previous cWCST studies [23].

### 2.3. Split-Half Reliability Estimation

For split-half reliability estimation, we considered systematic test splits as well as iteratively sampled random test splits [20,41,53]. Systematic test splits were obtained by splitting the total of a participants completed trials (1) into first and second halves and (2) into odd and even numbered trials. Sampling-based split-half reliability estimation comprised 1000 randomly assembled test splits. For any applied test split (i.e., first/second half split, odd/even split, and any random split), we computed the number of PE, SLE, and IE as well as mean RT on switch, repeat, and inference trials. For any of these performance indices, Pearson correlation coefficients *r* were computed between corresponding test halves and corrected for test length by the Spearman–Brown formula [51,52]

*r_SB_* = 2*r*/(1 + *r*) (1)

For sampling-based split-half reliability estimation, we summarized the frequency distribution of all sampled *r_SB_* by its median and the 95% highest density interval (HDI). The 95% HDI contains 95% percent of sampled split-half reliability estimates [20,53]. We used a modified variant of REL_EX_ for split-half reliability analysis [53]. That is, the modified version of REL_EX_ automatically computed means and standard deviations of conditional error probabilities and response times for any considered test half. REL_EX_ is a Microsoft Excel-based software tool for split-half reliability sampling. Split-half reliability analyses are available from https://www.osf.io/3ny95/.

## 3. Results

### 3.1. Descriptive Statistics

Table 1 shows descriptive statistics for the number of committed PE, SLE, and IE. Participants completed an average of 168.59 trials (*SD =* 14.38). About 83.12 (*SD* = 18.68) of these trials were switch trials on which participants committed an average of 12.16 PE (*SD* = 13.65). About 82.71 trials (*SD* = 8.30) were repeat trials. On repeat trials, participants committed an average of 5.03 SLE (*SD* = 7.07). Participants completed an average of 27.95 inference trials (*SD* = 8.30) with about 8.46 IE (*SD* = 8.82) committed.

For RT analysis, we excluded 0.03% of all trials because of a RT faster than 100 ms and we excluded 1.93% of all trials because of a RT slower than the individual cut-off. We also excluded those trials on which a PE, an SLE, or an IE was committed. Table 2 shows descriptive statistics for the number of remaining switch, repeat, and inference trials as well as for RT on these trial types. Remaining valid trials for RT analysis were about 68.76 switch trials (*SD* = 8.94), 77.41 repeat trials (*SD* = 13.18), and 18.98 inference trials (*SD* = 3.74). Mean RT were fastest on repeat trials (*m* = 1241 ms; *SD* = 441 ms), followed by inference trials (*m* = 1700 ms; *SD* = 636 ms) and switch trials (*m* = 1835 ms; *SD* = 694 ms).

### 3.2. Split-Half Reliability Estimation

**Figure 2:**
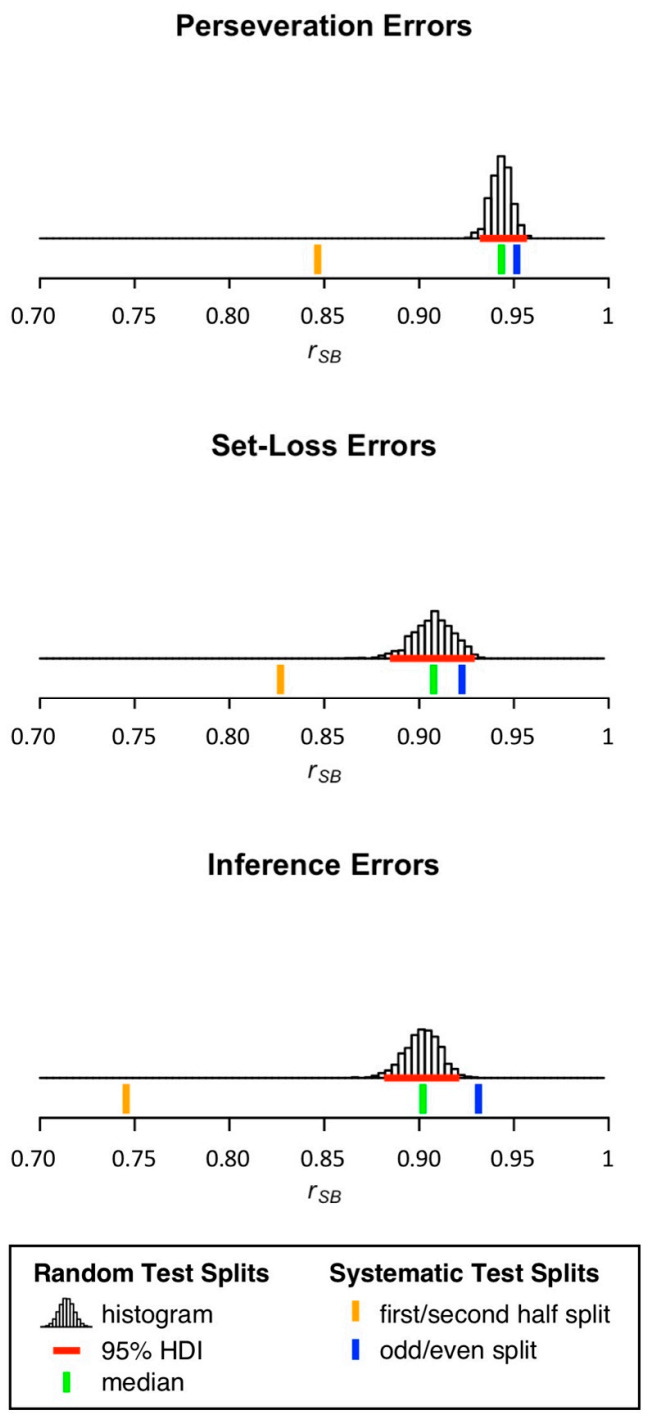
Distribution of split-half reliability estimates for the number of PE, SLE, and IE. 95% HDI = 95% highest density interval.

Table 1 and Figure 2 show split-half reliability estimates for the number of PE, SLE, and IE. For all error scores, medians of sampling-based split-half reliability estimation fell in the range between 0.90 < *r_SB_* < 0.95. The median of sampling-based split-half reliability estimation was highest for the number of PE, followed by the number of SLE, and the number of IE. Split-half reliability estimates obtained from first/second half splits were overall lower than medians of sampling-based split-half reliability estimation. In contrast, split-half reliability estimates obtained from odd/even splits were overall higher than medians of sampling-based split-half reliability estimation.

**Figure 3:**
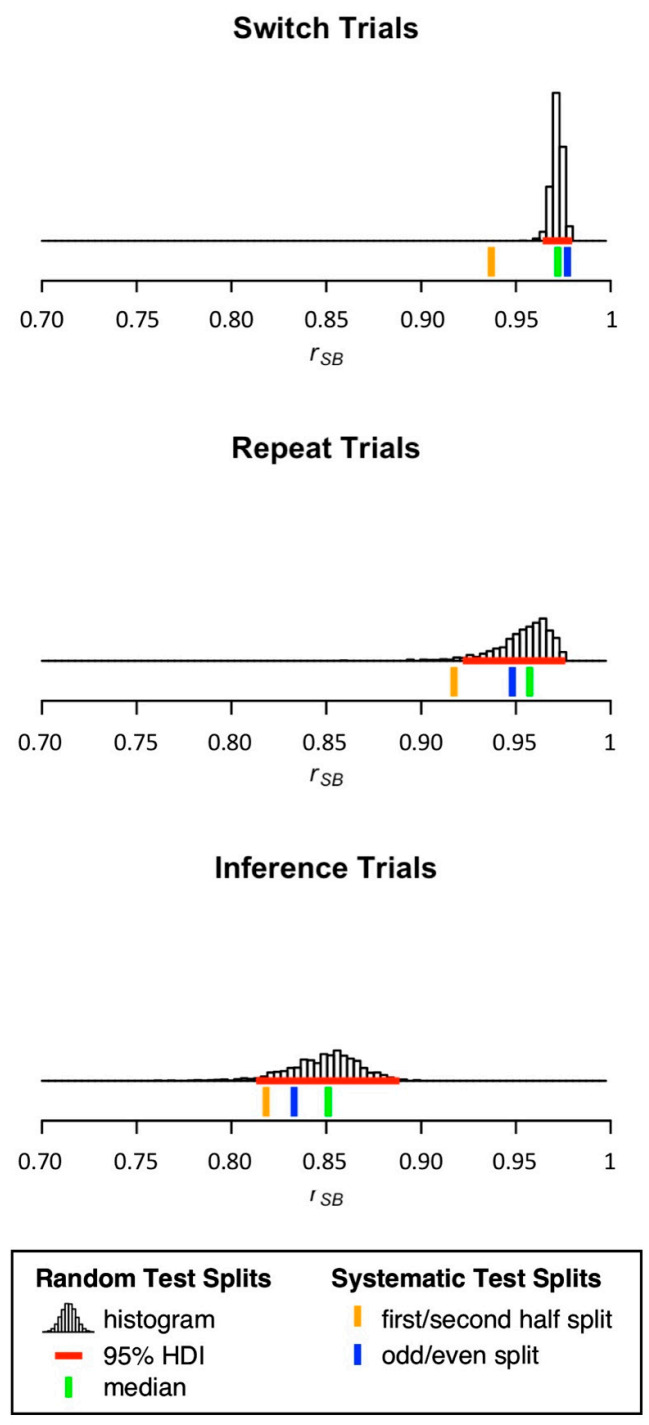
Distribution of split-half reliability estimates for mean RT on switch, repeat, and inference trials. 95% HDI = 95% highest density interval.

Table 2 and Figure 3 show split-half reliability estimates for mean RT on switch, repeat, and inference trials. For mean RT on switch and repeat trials, medians of sampling-based split-half reliability estimation were higher than 0.95. In contrast, the median of sampling-based split-half reliability estimation for mean RT on inference trials was considerably lower (*r_SB_* = 0.85). Again, split-half reliability estimates obtained from first/second half splits were overall lower than medians of sampling-based split-half reliability estimation. Only for mean RT on switch trials, the split-half reliability estimate obtained from the odd/even split was higher than the median of sampling-based split-half reliability estimation.

## 4. Discussion

The present study presents the first investigation of split-half reliability of a self-administered computerized WCST variant. Our analysis of cWCST data that were obtained from a large sample of young volunteers indicated overall high split-half reliability estimates for the number of PE, SLE, and IE: medians of sampling-based split-half reliability estimation of these measures fell in the range between 0.90 < *r_SB_* < 0.95. Moreover, medians of sampling-based split-half reliability estimation of mean RT on switch and repeat trials exceeded 0.95. Our results suggest that these cWCST measures provide adequate split-half reliability when self-administered in a sample of young volunteers.

Neuropsychological assessment’s reach is limited by its conventional administration set-ups that require face-to-face expert-administration of paper-and-pencil tests. Self-administered online assessment addresses these limitations by enabling examinees to remotely self-administer computerized assessment tools [6,7,8]. However, the practical implementation of self-administered online assessment requires the translation of well-established paper-and pencil assessment tools to digital format. Such translations to digital format typically necessitate changes in perceptual, cognitive, and/or motor features [2]. For example, manual WCST variants [21,26,27,28] present participants with concrete key and stimulus cards, whereas the cWCST presents participants with key and stimulus cards on a computer screen. On manual WCST variants, participants indicate their card sorts by placing stimulus cards next to key cards. In contrast, the cWCST requires participants to indicate card sorts by pressing one of four keys. On manual WCST variants, the examiner provides participants with verbal feedback, whereas the cWCST presents participants with visual feedback cues. These differences between manual WCST variants and the cWCST may impact reliability [2,5]. However, we found similar split-half reliability estimates for the cWCST as reported for a well-established manual WCST variant (i.e., the M-WCST [20]). For example, medians of sampling-based split-half reliability estimation for the number of PE was 0.94 for the cWCST and 0.92 for the M-WCST [20]. Thus, the named differences in test format between manual WCST variants and the cWCST do not seem to exert strong impact on split-half reliability.

The practical implementation of self-administered online assessment also requires the shift from face-to-face expert-administration to self-administration of assessment tools. On manual WCST variants, participants are provided with test instructions by the examiner. As participants are under constant supervision, the examiner may intervene if there are any (severe) deviations from the instructed behavior. In the present study, participants received cWCST instructions on the computer screen and were not supervised by the examiner. However, split-half reliability estimates for the self-administered cWCST were similar to split-half reliability estimates for the face-to-face expert-administered M-WCST [20]. Hence, self-administration of the cWCST when compared to conventional face-to-face expert-administration of the M-WCST seems to exert no strong impact on split-half reliability. It should be noted that we excluded around 8% of participants from the initial sample because of invalid cWCST performance (for details, see Data Collection). It remains to be shown whether participants with invalid cWCST performance misunderstood instructions or whether these participants were not committed to successfully perform on the cWCST. Future research is necessary to reduce the number of participants with invalid cWCST performance.

It should be highlighted that the reported split-half reliability estimates cannot be considered as an invariant property of the cWCST when self-administered. Instead, these split-half reliability estimates should be better conceived as a joint property of the cWCST when self-administered and the studied sample of young volunteers [65,66]. An important metric that determines reliability in that regard is the inter-individual variance of a sample, i.e., true differences between participants [67]. In general, a low inter-individual variance is associated with low (split-half) reliability estimates. Samples of young volunteers are likely to show a lower inter-individual variance (a more homogeneous performance as indicated by overall low numbers of PE, SLE, and IE) when compared to a sample of patients with various neurological diseases [20]. As a consequence, split-half reliability estimates are expected to be lower in studies of young volunteers when compared to studies of neurological patients. It therefore comes as a surprise that we found split-half reliability estimates of cWCST measures in a sample of young volunteers that are similar to those we had observed on the M-WCST in a sample of neurological patients [20].

This finding can be explained by the fact that the cWCST consists of more trials (*m* = 168.59 trials; *SD* = 14.38; in the present study) when compared to the M-WCST (a fixed number of 48 trials). Higher numbers of administered trials are associated with reduced measurement error, which increases reliability [51,52]. Thus, higher trial numbers of the cWCST may have been beneficial with regard to split-half reliability estimates, thereby counteracting potential adverse effects on split-half reliability of the purported low inter-individual variability of young volunteers.

We found generally lower split-half reliability estimates obtained from first/second half splits when compared to estimates obtained from splits by odd/even trial numbers. It has been argued that inter-individual long-term trends in performance (e.g., individual differences with regard to learning or fatigue) exert detrimental effects on split-half reliability estimates obtained from first/second half splits but not on estimates obtained from odd/even test splits [20]. For example, learning may improve one participant’s cWCST performance on the second test half (i.e., a reduction in the number of committed PE, SLE, and IE as well as faster RT), whereas another participant may show worse cWCST performance on the second test half because of fatigue. These individual long-term trends could decrease the correlation between performance indices on the first and second test half, reducing associated split-half reliability estimates. In contrast, individual long-term trends may impact split-half reliability estimates obtained from odd/even splits to a lesser degree, because such effects exert their influence on approximately as many odd as on even trials. Hence, our finding of lower split-half reliability estimates obtained from first/second half splits when compared to odd/even splits could be indicative of individual long-term trends in cWCST performance. 

The cWCST allows complementing the assessment of traditional error scores with RT measures, providing additional information about participants’ performance. For mean RT on switch and repeat trials, medians of sampling-based split-half reliability estimation exceeded 0.95. We found a relatively low median of sampling-based split-half reliability estimation for mean RT on inference trials (i.e., 0.85). This low split-half reliability estimate could be related to the relatively small number of inference trials that was entered into the RT analysis. Participants completed an average of 18.98 inference trials (*SD* = 3.74) compared to an average of 68.76 switch trials (*SD* = 8.94) and 77.41 repeat trials (*SD* = 13.18). These considerations suggest that the reliability of mean RT measures on inference trials should be improved by increasing the number of inference trials. How many trials are necessary to obtain sufficient reliability estimates should be investigated by a simulation study. Such a study could manipulate the number of trials entered into reliability analysis and investigate the resulting reliability estimates.

Our results suggest that the cWCST is appropriate for self-administration in samples of young volunteers. However, it remains to be shown whether it is also suitable for self-administration in populations that are typically examined in clinical practice, such as neurological patients. Future studies should investigate whether the utilized cWCST instructions are appropriate for successful self-administration in clinical samples. In addition, WCST variants that were tailored for administration in clinical practice incorporate relatively small numbers of trials [27]. Hence, the considerably higher trial number of the cWCST may be challenging for clinical samples. cWCST configurations with reduced trial numbers may be more appropriate for administration in clinical samples. However, a decreased trial number may reduce the reliability of cWCST measures, hence the trade-off between test length and applicability in clinical samples needs careful consideration in future work.

The present study addresses two important steps that are required for the practical implementation of self-administered online neuropsychological assessment. These steps are (1) the translation of traditional paper-and-pencil WCST variants to digital format (i.e., the cWCST) and (2) the switch from face-to-face expert-administration to self-administration. Our results suggest that these changes do not exert strong effects on split-half reliability of WCST measures. However, the practical implementation of self-administered online assessment requires further changes which might impact the (split-half) reliability of WCST measures. In the present study, participants self-administered the cWCST in the laboratory using identical computers. In self-administered online assessment, participants self-administer an assessment tool at their own homes using their own personal computers. Differences in computer characteristics (e.g., screen size or the operating system) or in testing environments (e.g., time of day or background noise) may introduce systematic variability in participants performance [2]. Hence, future research is necessary to study the effects of computer characteristics and testing environments on reliability (and validity) of the cWCST in self-administered online assessment [2]. We would like to highlight that the cWCST was programmed in OpenSesame [64], which allows easy switches to online administration by means of the open-source and freely available JATOS project [68].

## 5. Conclusions

We presented the first split-half reliability estimates for a self-administered computerized variant of the WCST. We found overall high split-half reliability estimates for error scores and most RT measures in a relatively large sample of young volunteers. Our results demonstrate that the considered cWCST measures show adequate split-half reliability when self-administered, paving the way for an advanced digital assessment of executive functions.

## Figures and Tables

**Table 1:** Descriptive statistics and split-half reliability estimates for the number of PE, SLE, and IE. *SD* = standard deviation; HDI = highest density interval.

	Number of Committed Errors		Split-Half Reliability Estimates				
			First/Second Half Split	Odd/Even Split	Random Test Splits		
					Median	95% HDI	
Error Type	Mean	SD				Lower	Upper
Perseveration Errors	12.16	13.65	0.8465	0.9515	0.9434	0.9318	0.9534
Set-Loss Errors	5.03	7.07	0.8269	0.9226	0.9076	0.8843	0.9259
Inference Errors	8.46	8.82	0.7455	0.9313	0.9020	0.8819	0.9185

**Table 2:** Descriptive statistics and split-half reliability estimates for RT measures. *SD* = standard deviation; HDI = highest density interval. Response times in milliseconds.

	Descriptive Statistics				Split-Half Reliability Estimates					
	Valid Trials		Response Time		First/Second Half Split	Odd/Even Split	Random Test Splits			
							Median	95% HDI		
Trial Type	Mean	*SD*	Mean	*SD*				Lower	Upper	
Switch Trial	68.76	8.94	1835	694	0.9370	0.9772	0.9721	0.9653	0.9772	
Repeat Trial	77.41	13.18	1241	441	0.9173	0.9481	0.9573	0.9190	0.9731	
Inference Trial	18.98	3.74	1700	636	0.8183	0.8330	0.8510	0.8059	0.8802	

## Data Availability

Data, split-half reliability analyses, the cWCST and test instructions are available from https://www.osf.io/3ny95/.
